# Tetanus

**Published:** 1860-06

**Authors:** H. Caruthers

**Affiliations:** Tarrytown


Tetanus.—*To the Editors of the Peninsular and Independent—Dear Sir:* I have been for some time a reader of your valuable Journal, and hope you will excuse the liberty I take in sending you an account of a case of Tetanus. In your number for December, 1859, I noticed the report of an interesting case in N. Y. Hospital, which called to mind the one I send.

October 28th, 1856. I was called to see a lad about nine years old, who had been injured by the wheel of a car passing over the foot, crushing and lacerating the parts sadly. The lower ends of both bones of the leg were splintered, which compelled the removal of the injured part, about the lower third.

The wound was brought together after securing the artery, and with a little sloughing of the injured parts all went on well; ligature came away; wound looked healthy. On the 16th of November I was summoned in haste at night to see the patient (in a fit, as stated by the messenger.) I found him with Tetanus, jaws locked, his tongue between the teeth badly cut, and his person covered with blood. His head and heel resting on the bed in a state of complete opisthotonos. I resorted to chloroform and ether, equal parts to relax the spasm, and ordered stimulants with beef tea, and commenced giving injections of assafœtida. This treatment was adhered to, and after four days of great anxiety and great suffering on the part of the little patient my efforts were successful in relieving him of all spasm. During this time the wound looked healthy; in truth, this fact urged me on. He was fed through a tube passed between his teeth, one having been removed for the purpose.

Truly and sincerely, yours, H. CARUTHERS.

*Tarrytown, January 26th*, 1860.

